# Determinants of patterns of maternal and child health service utilization in a rural community in south eastern Nigeria

**DOI:** 10.1186/s12913-017-2653-x

**Published:** 2017-11-13

**Authors:** C. C. Agunwa, I. E. Obi, A. C. Ndu, I. B. Omotowo, C. A. Idoko, A. K. Umeobieri, E. C. Aniwada

**Affiliations:** Department of Community Medicine, University of Nigeria/University of Nigeria Teaching Hospital, Ituku Ozalla Campus, Enugu, Enugu State Nigeria

**Keywords:** Maternal and child services, Utilization, Rural women, Factors

## Abstract

**Background:**

Women and children constitute a large proportion of any population. They are the most vulnerable to morbidity and mortality especially in developing countries. In many situations the problem of poor maternal and child health stems from the poor use of available services even when they are not of optimum quality. This study seeks to describe the patterns of utilization of Maternal and Child health (MCH) services in a rural area of Enugu State, and identify factors that are associated with and responsible for determining them.

**Methods:**

The study used a cross sectional analytic design. Pretested semi structured questionnaires were administered by interviewers to 602 women from a rural community in Enugu state, South east Nigeria. Two focus group discussions (FGDs) involving 8–10 men/ women each were conducted to identify factors affecting service utilization. Chi square analysis was done to identify factors associated with Maternal and Child Health services utilization. Logistic regression was used to identify determinants of utilization patterns. N vivo software was used to analyze findings of the FGDs.

**Results:**

The study revealed that increasing age, educational level, monthly income, number of children and occupation of both women and their husbands were associated with increased MCH service utilization. Average monthly income (OR: 1.317, *p* = 0.048, CI: 0.073–0.986) and number of children (OR: 1.196, *p* < 0.01,CI: 1.563–7.000) were determinants of increased use of child care services while educational level (OR: 0.495, *p* < 0.001, CI: 1.244–2.164) and age (OR: 0.115, *p* < 0.001, CI: 0.838–0.948) determined better use of delivery and family planning services respectively.

**Conclusions:**

Improved use of MCH services is related to socio economic challenges women face such as illiteracy and low income. Furthermore, the way health facilities and their staff are perceived by rural women affect how they use some of these services and should be considered in programs which seek to reduce maternal and child mortality.

Behavioral change programs with high local content need to be implemented within rural areas especially among younger, illiterate women .

## Background

Every year, more than 200 million women become pregnant, 15% of which are at risk of complications that have the potential to cause disease and death [1]. These risks exist in different societies and situations [2].

Persistently low utilization rates of Maternal and Child health (MCH) services in developing countries [3, 4], have led some authors to the conclusion that programs that rely only on ANC as a delivery system are likely to have poor coverage and compliance [5]. Alternative delivery approaches need to be identified as maternal and child mortality rates are unacceptably high in most developing countries, particularly in Sub Saharan Africa [6].

In Nigeria, only 38.1% of women receive skilled attendance at delivery and immunization coverage ranges between 43% and 16% in urban and rural areas [7] while 25% of children are fully immunized at age 23 months [7] in a country where the service has been almost completely free since its inception.

Continued ignorance about the factors that determine the health care choices women make for themselves and their children will lead to a continuation of the waste of already limited resources and already embarrassing mortality figures. Unless the factors responsible for observed service utilization patterns in rural areas of developing countries are identified it will be difficult to properly intervene to correct the discrepancy in health service supply and demand which occurs in Nigeria and other developing countries.

This study seeks to describe the patterns of utilization of maternal and child health (MCH) services (Family planning, Antenatal care, skilled delivery, child immunization and Child outpatient) in rural areas, and identify factors that are significantly associated with and responsible for determining them.

## Methods

This cross sectional analytic study was carried out in 2013 with 610 women of reproductive age who reside in rural communities of Enugu State. Only females aged 15–49 years who had resided for at least a year in the study area irrespective of their marital or gestational status who gave consent and available were included in the study. Ethical clearance was obtained from the Enugu State Research Ethics Committee at the State Ministry of health.

Multistage sampling technique was utilized to select the sample. In the first stage, one (Nkanu East) out of the 14 rural local government areas in Enugu State was selected. In the second stage, Nara community with a population of 10,446 was selected. Each stages of selection was carried out by the simple random sampling technique of balloting. Within the community, the number of houses recorded under the house numbering system, 1931 in number, was ascertained from the State headquarters of the National Population Commission. This was divided by the sample size (602) to give a sampling interval of 3. The index compound was selected also by balloting and subsequently, based on the sampling interval other compounds were selected. Within each compound women who met the inclusion criteria were interviewed. Where there were more than one eligible woman in the compound, one was selected by ballot.

Data was collected using an interviewer administered, semi structured questionnaire. The questionnaire was translated into the local language and pretested in a similar rural community, following which ambiguous questions were simplified. The questionnaire contained questions on the socio demographic characteristics of respondents, their level of utilization of the different MCH services, their reasons for using/not using them, and their experiences at the health center. They were administered by 4 trained interviewers; the average time of administration was 30 min. Data was checked at the end of each day by the authors and if any data was missing the interviewers returned to the household for it, where possible.

SPSS for windows version 18 (Chicago) was used for data entry and analysis. The MCH service utilizations of interest were attendance to antenatal clinic, delivery in a health facility, use of any family planning method, use of outpatient treatment of common childhood illnesses and uptake of immunization appropriate for child’s age (based on the national program on immunization). Descriptive analysis were expressed as percentages. Statistical testing was done using Chi square analysis and variables which had significant association with 1 or more service utilizations were entered into the regression model. Statistical significance was considered present at *p* value less than 0.05. Only respondents who were eligible for a MCH service were considered in calculation of utilization levels.

## Results

Table 1 shows that the categories of respondents with the greatest frequency were those aged 25–29 years; 160/602 (26.7%) with mean age (SD) of 27.2 years (6.7), those with secondary level of education 382/602 (63.7%), those who were married, 520/602 (86.4%), and unemployed; 242/602 (40.2%). The most prevalent occupation among their husbands was civil service; 174/602 (32.7%).

**Table 1:** Socio demographic characteristics of respondents

Variable	Freq (%)
Age group (years)	
15–19	108 (18.0)
20–24	124 (20.7)
25–29	160 (26.7)
30–34	124 (20.7)
35–39	54 (9.0)
40–44	20 (3.3)
≥ 45	10 (1.7)
Mean(SD*)	27.2 (6.66)
95% C.I.*	14.1–40.3
Range	15–49
Educational level	
None	12 (2.0)
Primary	72 (12.0)
Secondary	382 (63.7)
Tertiary	134 (22.4)
Marital status	
Married	520 (86.4)
Single	62 (10.3)
Widowed	20 (3.3)
Occupation:	
Unemployed	242 (40.2)
Trader	166 (27.6)
Civil servant	156 (25.9)
Artisan	20 (3.3)
Farmer	14 (2.3)
Student	4 (0.7)
Husband’s Occupation	*n* = 520
Civil Servant	174 (33.5)
Artisan	170 (32.7)
Trader	76 (14.6)
Farmer	38 (7.3)
Professional eg. Doctor, Lawyer	36 (6.9)
Pensioner	26 (0.1)

^*^ *CI Confidence interval, SD Standard deviation*

Table 2 shows most women had 1–4 children 404/ 602 (67.1%) and had a history of having ever lost a pregnancy 468/602 (77.7%).

**Table 2:** Respondents pregnancy history

Variable	Freq (%)
	*N* = 602
No of children	
None	74 (12.2)
1–4	404 (67.1)
> 4	124 (20.7)
Ever lost a pregnancy?	
Yes	468 (77.7)
No	134 (22.3)

Table 3 shows low level of use of family planning (FP) and institutional delivery (ID) across most age groups, especially in the youngest age groups between 15 and 24 years. There was significant association between age and use of all services except Child Out patient Department (COPD). (*p* < 0.0001 for family planning, ANC and Immunization and *p* = 0.001 for delivery services).

**Table 3:** Patterns of MCH service utilization among different age groups and levels of education

Variable	ANC Freq (%)		Imm. Freq (%)		ID Freq (%)		FP Freq (%)		COPD Freq (%)	
	T	*n* = 504	T	*n* = 528	T	*n* = 464	T	*n* = 602	T	*n* = 512
Age group (years)										
15–19	16	12 (75.0)	20	8 (40.0)	0	0 (0.0)	44	2 (4.5)	10	6 (60.0)
20–24	34	34 (100.0)	36	36 100.0)	52	12 (11.5)	56	2 (3.6)	36	34 (94.4)
25–29	116	116 (100.0)	124	112 (90.3)	124	56 (22.6)	136	16 (11.8)	120	110 (91.7)
30–34	144	142 (98.6)	148	142 (95.9)	144	88 (30.6)	160	30 (18.8)	148	140 (94.6)
35–39	108	104 (96.3)	114	106 (93.0)	104	22 (21.2)	116	44 (37.9)	112	104 (92.9)
40–44	56	56 (100.0)	56	52 (92.9)	56	26 (46.4)	60	34 (56.7)	56	46 (82.1)
≥ 45	30	30 (100.0)	30	30 (100.0)	26	10 (38.5)	30	20 (66.7)	30	28 (93.3)
X^2^		25.132		41.730f		21.864f		55.483f		10.732f
Df		6		6		6		6		6
*p*-value		<0.0001		<0.0001		0.001		<0.0001		0.097
Level of education										
None	4	2 (50.0)	2	2 (100.0)	0	0 (0.0)	0	0 (0.0)	0	0 (0.0)
Primary	86	72 (83.7)	90	88 (97.8)	92	20 (21.7)	96	34 (35.4)	0	0 (0.0)
Secondary	288	242 (84.1)	270	268 (99.2)	286	60 (21.0)	328	68 (20.7)	280	34 (12.1)
Tertiary	162	128 (79.0)	136	134 (98.5)	146	66 (45.2)	174	46 (26.4)	144	10 (6.9)
X^2^		3.341f		3.874f		16.158f		11.365f		7.594f
Df		5		5		5		5		5
*p*-value		0.648		0.568		0.006		0.045		0.180

Where, *n* number of women using MCH service, *T* total number of women in the group

There was higher level of use of family planning, child outpatient (OPD) and institutional delivery services among educated women compared with non-literate women. There was also better utilization of ANC, immunization and delivery services (476/602; 79.0%, 593/602; 98.5% and 272/602; 45.2% respectively) among women with tertiary education when compared with their other educated contemporaries. Family planning use was generally poor among all respondents. There was significant association between educational level and use of delivery (*p* < 0.001) and family planning (*p* = 0.05) services.

Table 4 reveals a high level of use of 100% in ANC, Immunization and COPD services among farmers but less of Family planning services (28.6%) and none reported to have utilized institutional delivery services. Also worthy of note was the fact that unemployed women consistently had one of the lowest utilization figures and civil servants had consistently average to high rates compared to other groups. A significant association was found between occupation and the use of ANC (*p* = 0.02) and institutional delivery services (*P* = 0.03).

**Table 4:** Utilization of MCH services among different occupational groups

Variable	ANC Freq (%)		Imm. Freq (%)		ID Freq (%)		FP Freq (%)		COPD Freq (%)	
	T	*n* = 504	T	*n* = 528	T	*n* = 464	T	*n* = 602	T	*n* = 512
Respondents occupation										
Unemployed	242	178 (73.6)	182	178 (97.8)	242	46 (19.0)	242	42 (17.4)	194	178 (91.8)
Trader	166	152 (91.6)	150	150 (100.0)	166	48 (28.9)	166	50 (30.1)	154	138 (89.6)
C/servant	80	130 (83.3)	132	130 (98.5)	156	46 (29.5)	156	48 (30.8)	136	130 (95.6)
Artisan	20	16 (80.0)	16	16 (100.0)	20	4 (20.0)	20	2 (10.0)	18	14 (77.8)
Farmer	14	14 (100.0)	14	14 (100.0)	0	0 (0.0)	14	4 (28.6)	14	14 (100.0)
Student	4	4 (100.0)	4	4 (100.0)	4	4 (100.0)	4	2 (50.0)	4	2 (50.0)
X^2^		13.124f		1.929f		12.411f		8.296f		9.118f
Df		5		5		5		5		5
*p*-value		0.022		0.859		0.030		0.141		0.104
Husbands Occupation										
Unemployed	0	0 (0.0)	0	0 (0.0)	0	0 (0.0)	0	0 (0.0)	0	0 (0.0)
Trader	48	46 (95.8)	48	48 (100.0)	50	18 (36.0)	50	16 (32.0)	48	40 (83.3)
C/servant	138	136 (98.6)	134	132 (98.5)	114	34 (30.0)	148	46 (31.1)	140	108 (77.1)
Artisan	134	132 (98.5)	134	130 (97.0)	146	28 (19.2)	146	46 (31.5)	138	112 (81.2)
Farmer	12	12 (100.0)	12	12 (100.0)	14	6 (42.9)	12	2 (16.7)	14	10 (71.4)
Self employed	82	82 (100.0)	82	82 (100.0)	84	20 (23.8)	84	16 (19.0)	84	70 (83.3)
Professional	12	10(83.3)	12	12(100.0)	12	8 (66.7)	12	2 (16.7)	12	10 (83.3)
X^2^		13.751f		2.407f		23.889f		9.474f		59.115f
Df		7		7		7		7		7
*P* value		0.088		0.966		0.002		0.304		0.026

Where, *n* number of women using MCH service, *T* total number of women in the group, *C/servant* civil servant

Table 4 also shows use of ANC and immunization services (100%) among women whose husbands were farmers. There are generally high utilization rates for all services except family planning and delivery services. It also shows significant association between husbands’ occupation and the use of institutional delivery and COPD services. (*P* < 0.001 and *p* = 0.026 respectively).

As shown in Table 5, a chi square analysis revealed that among those who used delivery and family planning services, the proportion of users was greatest in the groups which had good assessments of the local health facility and lowest among those that scored the facility “poor”. This perception by the women was significantly associated with their utilization of delivery (*p* = 0.010) and family planning services (*p* = 0.047).

**Table 5:** Relationship between perception of the facility and MCH service utilization

Assessment of health center/ health staff	Utilization of institutional delivery services		Utilization of family planning services	
	Yes	No	Yes	No
	F (%)	F (%)	F (%)	F (%)
Poor (0–6) *n* = 532	240 (45.1)	292 (54.9)	91 (17.1)	441 (82.9)
Good (7-13) *n* = 70	55 (78.6)	15 (21.4)	18 (25.7)	52 (74.3)
X^2^	15.924		7.964	
*P* value	0.014		0.047	

In Table 6, logistic regression identified average monthly income (OR: 1.317, *p* = 0.048, CI: 0.073–0.986) and number of children (OR: 1.196, *p* < 0.01, CI: 1.563–7.000) as determinants of increased use of child outpatient care. Educational level (OR: 0.495, *p* < 0.001, CI: 1.244–2.164) was a determinant of increased use of delivery services and age (OR: 0.115, *p* < 0.001, CI: 0.838–0.948) was a determinant of increased use of family planning services.

**Table 6:** Significant determinants of MCH service utilization

MCH service	Variable	OR	*P* value	95% C.I.	
				Lower limit	Upper limit
COPD	Ag. Monthly income				
	<N 10,000	1			
	>100,000	1.317	0.048	0.073	0.986
	No of living children				
	1–4	1			
	>4	1.196	0.002	1.563	7.000
Delivery	Educational level				
	Is not educated	1			
	Has tertiary education	0.495	0.000	1.244	2.164
FP	Age				
	Age < 20 yrs	1			
	Age 40–44 yrs	0.115	0.000	0.838	0.948

*COPD* child outpatient department

## Discussion

Utilization of antenatal care (ANC) and immunization services for children were generally high with peak values in older women of 40 years and above and the lowest utilization rates in the 15–19 year age group. Women aged between 25 and 34 years were more likely to use antenatal, delivery, family planning and immunization services than those younger than 25 years with the lowest utilization rates found among women who were less than 20 years of age. Institutional delivery and family planning utilization were generally low, reaching its peak utilization rate among women older than 40 years. This may be attributed to their being more experienced and more enlightened in childbearing and child care activities. Also women are usually better empowered financially by this age giving them more freedom to take decisions and make personal choices they believe are in their best interest [8, 9]. Age was identified as a determinant of family planning services among these women.

The utilization of family planning services was common among the educated groups of respondents. The consistently high MCH utilization rates among farmers is worthy of note and differs from findings in Malaysia where women whose husbands worked in business/service were more likely to seek treatment from a doctor or nurse compared with wives of farmers (*p* < 0.01) [10]. Utilization rates were highest among civil servants and consistently low among the unemployed. This supports previous findings about the influence of financial barriers on utilization of MCH services in developing countries [11].

Utilization of Institutional delivery services increased with and was found to have a significant association with husband’s occupation reaching its highest rates where the husband was a professional or farmer. Significant association of husband’s occupation with use of family planning and immunization services was also observed. The reason for this may be that in this part of the world where husbands tend to be sole decision makers and in which there is financial dependence on them by their wives, they have a strong influence on the health service utilization practices of their wives and so women with educated and/or wealthy husbands are better supported to adopt proper health seeking behaviors [10].

The study found, once again, an increase in the utilization of ANC, child care and delivery services with increasing level of education of the women themselves up to a peak among those with tertiary school education, and then a decline among non-literate women. Education was also identified as a factor determining use of delivery services. A very similar pattern is seen in the utilization of Immunization, Family Planning and Institutional Delivery services and women without any formal education consistently had the lowest utilization rates for every service.

Also, there was significant association observed between education and use of all MCH services except OPD services. This pattern is similar to the findings of other authors [12] and may be due to the fact that with educational attainment comes increased awareness of health related information which predisposes one to better utilization of health services [13]. However, a limitation of the study may be the fact that some categories of education and occupation had no observations. Further studies may be required to support findings related to these characteristics.

What this implies is that the higher the literacy rate of any population the higher the rate of utilization of MCH services. There is therefore need to ensure access of women to basic education in order to increase national literacy rates in developing countries. The exception noticed in COPD utilization may be due to higher patronage of other non- formal care providers such as patent medicine vendors and chemists who are perceived as legitimate care givers by majority of society [14].

Increasing age and education level were associated with better utilization of immunization services while the reverse was the case with increasing monthly income and occupation, and surprisingly, number of children.

The use of child care (outpatient and immunization) services increased with income but decreased with age. Delivery at formal institutions improved with better education, occupation and income earned supporting the idea that finance plays a role in choices made when deciding whether or not to utilize health services.

Age was found to be a significant determinant of family planning use with very poor patronage among women less than 20 years of age and increasing service utilization in subsequent older age groups. This supports the findings of the FGD which revealed the perception, especially among younger women, that family planning was meant only for women who had completed their families.

Occupation of women was found to have significant effect on service utilization. This is in keeping with findings of studies carried out in the Philippines [15] and Ghana [16] in which occupation was found to strongly influence use of maternal services with civil servants and other professionals found to be more likely to use MCH services. Nevertheless, contrasting findings have been reported in Kuwait [17] where the possession of a paying job was found not to have any influence on the use of Family planning and other MCH services by women. However the differences in the religious and cultural backgrounds for these studies may account for the differences in the results seen because in societies where gender equity and empowerment are not taken seriously, the ability of the educated woman to make health related choices and pay for services is still significantly constrained.

Use of these services generally increased with increasing number of pregnancies except use of child OPD services. This differs from the findings of other studies [18, 19] in which the proportion of women most likely to utilize maternal services decreased with increasing birth order. This may be due to increased confidence in health services with each successful delivery and is actually a welcome development considering the higher risk of maternal morbidity and mortality associated with increasing numbers of pregnancies.

Although the use of maternal and child services was found to be associated with the social classes of these women represented by average income, occupation and education, none of these factors proved to be a significant determinant of service use. Another study [20] also shows differences in the utilization rates for women in different socioeconomic levels, with women from lower levels being less likely to seek orthodox health care services than their counterparts from higher socioeconomic strata. It is also important to note that average monthly income which can be difficult to estimate in self-employed individuals, may not be the best index of socio economic status and other indices such as property owned and education achieved may need to be used in future studies to further support these findings.

The utilization of maternal and child services was also found to be dependent on the perception of the health center/health staff by members of the community. A statistically significant association was identified between perception of the health facility and utilization of delivery and family planning services. This is similar to the findings of studies in Uganda [8], and the Dominican Republic [21]. In the Ugandan study, members of a tribe called “Alurs” do not usually attend ANC or deliver in the health center because of the embarrassment involved in being considered by other community members as being too weak to deliver without the assistance of a midwife. During this study, an informal interview with the Officer in charge of the health center revealed that a major factor which discouraged service use in the area was the cultural belief that women who delivered in the health center had been unfaithful to their husbands.

Women mentioned caring attitude of health workers as the major factor, affecting their patronage/non patronage of the health center. This was much more than those who considered the quality of care or even cost. Similar findings were observed in Bangladesh [22], Yemen [23] and studies in Nigeria and other African countries [24, 25] where underutilization of maternal and child services was attributed to the poor behavior of staff and preference for a more friendly atmosphere.

## Conclusion

Key characteristics of rural women which play major roles in influencing their use of these services are age, education, number of children, occupation, income and their perception of health workers and the facility. The poor levels of utilization among women less than 20 years give cause for concern. It identifies them as a major target for future interventions aimed at achieving a change in attitude and subsequently an improvement in MCH service use. The influence of respondents’ husbands on their use of services makes a strong case for increased male involvement in delivery of MCH services.

The provision of adequate numbers of properly trained health workers at well-equipped health facilities is essential, and if implemented will improve use of available services and contribute to reduction in maternal and child mortality rates.

## Abbreviations

ANC: Antenatal Clinic
CI: Confidence Interval
FGD: Focus Group Discussion
FP: Family Planning
ID: Institutional Delivery
MCH: Maternal and Child Health
OPD: Out Patient Department
OR: Odds ratio
SD: Standard Deviation
SPSS: Statistical Package for the Social Sciences
